# Human amniotic membranes as an allogenic biological dressing for the treatment of burn wounds: Protocol for a randomized-controlled study

**DOI:** 10.1016/j.conctc.2023.101209

**Published:** 2023-09-16

**Authors:** Pablo Pfister, Pedro David Wendel-Garcia, Isabelle Meneau, Mauro Vasella, Jennifer Ashley Watson, Philipp Bühler, Daniel Rittirsch, Nicole Lindenblatt, Bong-Sung Kim

**Affiliations:** a Department of Plastic Surgery and Hand Surgery, Burn Center, University Hospital Zurich, Zurich, Switzerland; b Department of Intensive Care, Burn Center, University Hospital Zurich, Zurich, Switzerland; c Department of Ophtalmology, Eye Bank Laboratory, University Hospital Zurich, Zurich, Switzerland; d Department of Intensive Care, Cantonal Hospital Winterthur, Winterthur, Switzerland

**Keywords:** Amniotic membranes, Burns, Burn wounds, Skin graft, Wound healing

## Abstract

**Background:**

Burn wounds pose significant challenges in medical treatment due to their devastating nature and resource-intensive requirements. Temporary coverage of burn wounds using synthetic or biological dressings allows for reepithelization before definitive skin grafting. Allogenic skin grafts have been widely used but come with drawbacks such as rejection and disease transmission. The use of amniotic membranes (AMs) offers a promising alternative for temporary coverage, as they possess biological properties that promote faster healing and improved scar quality. The various components of the amniotic membrane, including pluripotent stem cells, extracellular matrix proteins, and regenerative factors, contribute to cell growth, migration, and differentiation, as well as preservation of the original epithelial phenotype.

**Objective:**

Reliable information on the treatment of burn wounds with AM is needed. The knowledge gained in this project may help to include this advantageous modern concept of biological dressings in clinical practice. The purpose of this study is to use human amniotic membranes from our in hospital laboratory, as an allogenic biological dressing after enzymatic debridement in superficial partial thickness, deep partial thickness or full thickness burn wounds.

**Methods:**

We will include 30 patients in a randomized-controlled trial with each patient receiving the study intervention and the control intervention. Two 7 × 7 cm burn wound areas will be compared regarding percentage of skin graft take, healing time, healing percentage value and total healing time. Human amniotic membranes will be compared to allogenic skin grafts.

## 1 Introduction

### 1.1 Background and rationale

The treatment of burn wounds remains one of the most challenging problems in medicine, since burns are amongst the most devastating forms of injury and require a substantial number of resources [1,2]. The cornerstones of burn treatment are early debridement and subsequent skin grafting [3]. Synthetic or biological dressings are used after successful debridement, to cover the burn wounds, until definitive skin grafting is possible [4,5]. This temporary coverage of the wound bed, gives the tissue time for reepithelization as a preparation until definitive coverage can be achieved with skin grafts [6]. Allogenic skin grafts have been widely accepted in the surgical burn care [[7], [8], [9], [10]]. Potential disadvantages of allograft use include rejection und disease transmission [11]. Skin rejection is likely to occur within 2 weeks after application and is considered inevitable [9,12]. The numerous dendritic cells play a key role in the rejection mechanism and making allogenic skin one of the most immunogenic tissues [13]. As it is our only supplier, we strongly depend on the ETB-BISLIFE non-profit-organization, and we already had to face a supply shortage in the past. Furthermore, the high cost of allogenic donor skin plays an important role in the economic burden of burn care

The use of amniotic membranes (AMs) represents an innovative approach to the, up to now, unsatisfactory temporary coverage of burn wounds. Due to the biological properties of the amniotic membrane, faster healing and better scar quality could be achieved [2,4,14]. Several mechanisms have been suggested to explain the unique beneficial effects of amniotic membrane [15]. The main structure of the amniotic membrane consists of a multi-layered membrane, each of them containing different key components: among them the epithelium, which is considered as a reservoir of biological active pluripotent stem cells; the basal membrane made of extracellular-matrix proteins such as collagen and fibronectin; the stroma and the spongy layer which both contain various important regenerative factors and molecules [16]. These components have been shown to induce cell growth, migration, and differentiation of epithelial cells, and to support preservation of the original epithelial phenotype. In addition, amniotic membrane exhibits a low or even no antigenicity [15,[17], [18], [19], [20]]. The principal mechanism of effect of amniotic membrane on wound healing is the recruitment and engraftment of endogenous progenitor cells stimulating neovascularization and wound repair [21,22]. Furthermore, AM attenuates the local inflammatory response due to the secretion of chemokines and cytokines, involved in the regulation of the immunologic response at the wound site [23]. Thus, AM can positively influence the three phases of wound repair: inflammation, angiogenesis, and production and remodeling of the extracellular matrix [23,24].

Preclinical studies in the murine model have shown that the use of AM inhibits local inflammation, stimulates cell regeneration, and furthermore stimulates collagen production [25]. In addition, the application of AM was associated with decreasing the bacterial count in infected rat burn wounds [26]. In the ovine model, Fraser et al. showed a significant reduction of the amount of scar tissue generated after treatment of standardized burn wounds with AM [27].

Amniotic membranes have been used for decades in the treatment of burn wounds, with its first use reported in 1912 [27,28]. Ever since, several studies have shown the numerous beneficial of the use of the amniotic membranes and its impact in the wound healing process: pain relief, reduction of the inflammation, control of the water loss, diminution of the bacterial colonization, prevention of the scar formation and promotion of the epithelization and wound healing in burn wounds [[29], [30], [31], [32], [33]]. Additional advantages include easy handling, light weight, elasticity, adhesiveness, semipermeable properties and low immunogenicity [2,29,33].

In a systematic review conducted by Yang et al., in 2021, burn patients treated with amniotic membranes showed a significant reduction of bacterial invasion, a smaller number of dressing changes needed, a reduction of itching and pain and overall shorter healing times compared to conventional methods [30].

Over the past decades, the use of AM has expanded into various fields of medicine: in ophthalmology AM is increasingly used for ocular surface reconstruction in a variety of ocular pathologies [34]. Furthermore, the use of amniotic membranes has shown increased rates and shorter durations in wound healing in difficult-to-heal fistulas, diabetic and venous ulcers [[35], [36], [37], [38]].

### 1.2 Study objectives

The purpose of this study is to use human amniotic membranes from our hospital eye bank laboratory, as an allogenic biological dressing after enzymatic debridement in superficial partial thickness, deep partial thickness, or full thickness burn wounds. Our long-term goal is the implementation of the treatment with amniotic membranes as a standard of care with an in-hospital supply chain. We believe that this will provide a clinically and economically alternative to allogenic skin grafts.

This study aims to show that amniotic membranes are a safe and alternative allogenic biological dressing for the treatment of enzymatically debrided burns with superior rates of autologous skin graft take percentage when compared to allogenic skin grafts. Secondary objectives are to assess effects of the treatment with amniotic membranes regarding healing percentage value, healing time, and incidence of hypertrophic scar formation.

## 2 Materials and methods

### 2.1 Study design

**Fig. 1:**
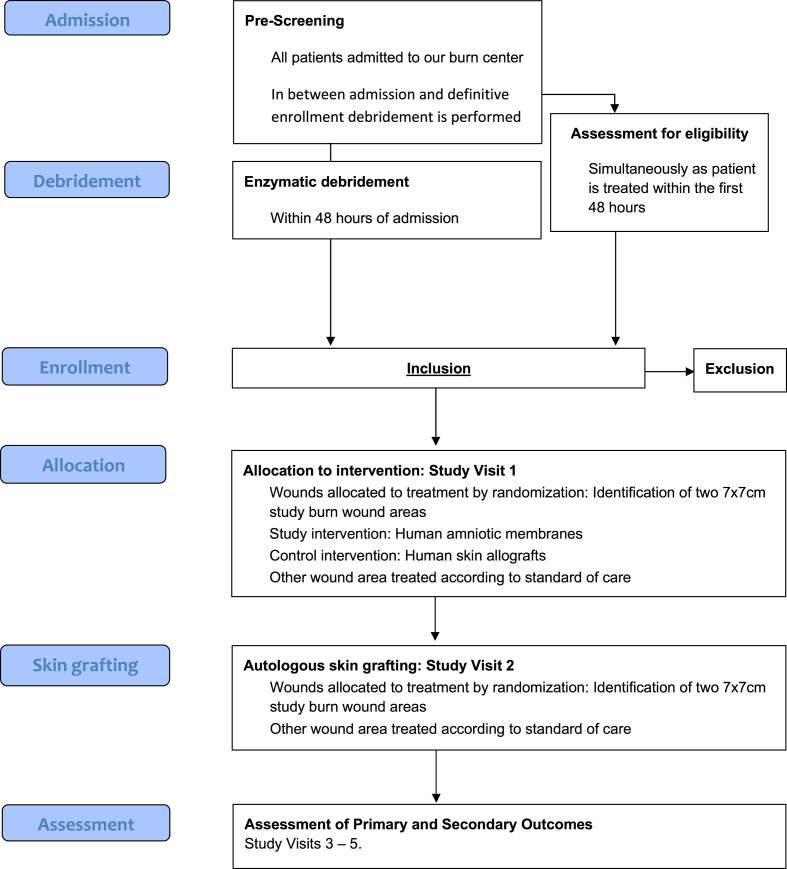
CONSORT-diagram.

The study is a randomized controlled trial investigating human amniotic membranes as biological wound dressings in the treatment of superficial partial thickness, deep partial thickness or full thickness burn wounds of the upper extremities, the torso or the lower extremities requiring enzymatic debridement. This intervention will be compared to human skin allografts. As a study population all burn patients admitted to our burn unit will be screened at admission. A total of 30 patients will be included in this study. The total duration for an individual patient will be 92 days from the admission to our burn unit to the 3-month postoperative control. See Fig. 1 for the CONSORT-diagram.

### 2.2 Study setting

A total of 30 patients will be included in this study during a study period of 2 years. The study will take place in the burn center of the University Hospital of Zurich in a highly specialized and interdisciplinary team. All patients treated in our burn center will be pre-screened upon admission for possible inclusion into our study.

### Ethics approval

The study will be carried out in accordance with principles enunciated in the current version of the Declaration of Helsinki, the guidelines of Good Clinical Practice (GCP) issued by ICH, and Swiss competent authority's requirements. The ethics committee of the Canton Zurich approved the study protocol (Kantonale Ethikkommission Zurich BASEC ID 2023-00457). The study will be registered on ClinicalTrials.gov before screening start.

### 2.3 Eligibility

Participants fulfilling all the following inclusion criteria are eligible for the study.

• Patients with mixed or deep partial thickness degree burns of the upper extremities, the torso or the lower extremities requiring enzymatic debridement
• Two burn areas of 7 × 7cm are available for study inclusion
• The patient is ≥ 18 years of age on day of admission
• Documented informed consent (IC) by signature or in case of emergency situations confirmation of a physician not involved in the study according to HFG Art. 30
The presence of any one of the following exclusion criteria will lead to exclusion of the participant:
• Patients with hepatitis B or C, syphilis or HIV positivity
• Patients with known underlying or concomitant medical conditions that may interfere with normal wound healing (systemic skin and connective tissue diseases, Cushing syndrome or disease, scurvy, chronic hypothyroidism, congenital or acquired immunosuppressive condition, chronic renal failure, or chronic hepatic dysfunction, severe malnutrition)
• Concomitant illness which, in the opinion of the Investigator, has the potential to significantly delay wound healing
• Severe drug or alcohol abuse
• Pre-existing coagulation disorders
• Enrolment of the Investigator, his/her family members, employees or other dependent persons
• Wounds in the study target area other than burn wounds
• Patients with a transplantation in the past medical history
• Delayed admission: time from injury to admission >24 h
• Pregnant or lactating females (blood tests for pregnancy will be done on all women of childbearing age after local standard of practice)
• Life expectancy of less than 72 h
• In case of emergency situations: visible opposition to participate in the research practice, expressed either verbally or through behavior

### 2.4 Experimental intervention: human amniotic membrane

The cryoconserved human amniotic membranes are already prepared in the eye bank from the University Hospital of Zurich using a standardized procedure according to Good Manufacturing Practice (GMP) and are used for transplantation in ophthalmologic procedures. The preparation of the amniotic membranes is well established and are defined in a standard operating procedure (SOP), implemented by the laboratory bank. Potential placental donations are identified from patients undergoing elective cesarean section in our hospital department of obstetrics and gynecology. After harvesting a rigorous screening process including donors, recipients and tissues is applied to ensure safety. After final processing, the amniotic membranes are put on 7 × 7cm Epigard® foil with the epithelium facing down towards the scaffold.

#### 2.4.1 Serological screening tests of the potential donor

For serological screening tests blood samples should be obtained at the time of donation or, if this is not possible, within 7 days before or 7 days after donation. In addition, a repeat sampling usually required after 180 days for the living donor, can be waived if in addition to the serological tests, the HIV, HBV and HCV viral genomes could be determined using an appropriate molecular nucleic acid amplification (i.e., polymerase chain reaction (PCR)).

In the Eye Bank, after informed consent is obtained from the donor, the following tests are performed on blood sample taken either the day prior or on the day of the planned cesarean section.

• HIV: HIV Antigen/Antibody Combo (Screening), HIV-RNS (PCR)
• Hepatitis C: anti HCV (Screening), HCV-RNS (PCR)
• Hepatitis B: HBs-Ag, *anti*-HBs (quant), *anti*-HBC-IgG/IgM, HBV-DNS (PCR)
• HTLV 1/2
• Lues: *Treponema pallidum* Particle Agglutination (TPPA)

#### 2.4.2 Microbiological testing on placenta and amnion membrane

During the processing of the amniotic membranes, samples for detecting aerobic and anaerobic bacteria and fungi must be obtained from the transport medium, from the different washings of the amniotic membrane and from piece of tissue before and after processing. Thus, several microbiological, mycological and molecularbiological tests are performed as follows:

Donated native placenta.

• *Chlamydia trachomatis* (PCR) using Cobas® PCR Media Dual Swab
• Mycoplasma genitalium (PCR) from Cobas® PCR Media Dual swab
• Neisseria gonorrhea (PCR) from Cobas® PCR Media Dual swab

Processed tissue.

• Mycological studies: direct microscopy, mycological culture on different selective culture medium and Periodic Acid-Schiff (PAS) staining using a COPAN® swab
• Bacteriological studies: Gram staining microscopy and anaerobic/aerobic on different selective culture medium using a COPAN® swab

### 2.5 Control intervention: human skin allograft

Glycerol-Preserved Allograft provided by the ETB-BISLIFE foundation in the Netherlands. This is an approved transplant product for the use in burn wounds and is our burn centers standard of care. Our burn center has a stock of allografts that is continuously updated.

### 2.6 Concomitant interventions (treatments)

Following concomitant interventions will be performed but are not considered study specific.

• Enzymatic debridement with Nexobrid® (Mediwound Ltd, Israel)
• Autologous skin grafting and transplantation. This will be performed according to in hospital standard operating procedures as this is a regular surgical intervention in burned patients. The timing of the operation will be from days 3–14, depending on the wound area. There is no specification with regards to the processing of split thickness skin grafts. Split thickness skin grafts can be meshed (ratio range from 1:1.5 to 1:6 usually) or unmeshed. Importantly, the experimental and control area are eventually treated with the same processed split thickness skin graft. The assessment is multifactorial and very much depends on the expertise of the burn surgeon. It is of paramount importance for an experienced attending surgeon to evaluate the wound. The donor site will be chosen according to the available sites by the corresponding plastic surgeon.

## 3 Study outcomes

### 3.1 Primary outcome

The primary outcome will be an assessment of the percentage of skin graft take. This main result will be reported as a percentage value in relation to the respective treated area.

This will be assessed during study visits 3 and 4, which will be performed by the principal investigator or investigator from the department of plastic surgery and will be documented using photographs.

### 3.2 Secondary outcome

Following secondary outcomes will be measured.

• Healing time: time to complete healing measured in days and assessed on study visits 3–5 which will be performed by the principal investigator or investigator from the department of plastic surgery and will be documented using photographs.
• Scarring: Pathological scarring such as hypertrophic scars, keloids, scar contracture or other aspects such as hypo- or hyperpigmentation are documented three months postoperatively on study visit 5 from the documented photographs. The further analysis will be performed by the principal investigator or his designee from the department of plastic surgery. The Patient and Observer Scar Assessment Scale (POSAS) will be used to objectify this outcome [39].
• Healing percentage value: Assessment of successful wound healing, either as a preparation for skin grafting or also when no skin graft was applied. This main result will be reported as a healing (epithelialization) percentage value in relation to the treated area. This will be assessed during study visits 2, 3 and 4 which will be performed by the principal investigator or investigator from the department of plastic surgery and will be documented using photographs.

### 3.3 Safety outcomes

Viral/bacterial disease transmission: there will not be a routine serological follow-up test after the initial screening laboratory. In any case of suspicion of a possible disease transmission thorough follow-up testing will be performed in coordination with the Sponsor-Investigator. These follow-up tests will include, but are not limited to.

• Human Immunodeficiency Virus (HIV): HIV Ag/Ab Combo (Screening), HIV-RNS (PCR)
• Hepatitis C (HCV): *anti*-HCV (Screening), HCV-RNS (PCR)
• Hepatitis B (HBV): HBs-Ag, *anti*-HBs (quant), *anti*-HBC-IgG/IgM, HBV-DNS (PCR)
• Human T-Lymphotropic Virus 1/2
• Lues: *Treponema pallidum* particle agglutination

## 4 Study plan

### 4.1 Screening/admission

According to standard operating procedures in our burn center, patients receive initial enzymatic debridement if they have mixed- or deep-partial thickness burns and this intervention is not considered study-specific. The usual timeline from admission to debridement is 48 h. Approximately 48 h after enzymatic debridement, the coverage with biological dressings takes place. This gives us a timeline from admission to the first study specific intervention of approximately 48–96 h. It is important to recognize that time points may depend on patient- and organizational-specific aspects. Within this time period following will be performed.

• Informed consent by signature or by inclusion of an independent physician. This will be done by the attending or resident burn surgeon on call. The participant will be given a minimum of 12 h from information to definitive consent. Given the urgency of treatment in burns, in some cases included patients will not be able to consent personally to the inclusion of this study. We will apply strict inclusion/exclusion criteria and informed consent procedures to guarantee ethical conduct of this study.
• Extraction of the routinely documented demographic data, medical history and burn history.
• Extraction of the laboratory parameters on admission.
• Extraction of the vital parameters on admission.

### 4.2 Enzymatic debridement

The enzymatic debridement will take place within 48 h after admission and is not considered a study-specific intervention. This intervention will take place according to standard operating procedures and will be performed by the attending or resident surgeon on call.

### 4.3 Study visit 1: Selection of study burn areas/randomization/intervention

For each patient two 7 × 7cm superficial partial thickness, deep partial thickness or full thickness burn areas with similar burn depths will be compared and allocated to their respective treatment arm by randomization. The randomization will be stratified for wound size. Every patient serves as their own control in the sense that only patients will be included with at least two wounds, of which one wound is treated with amniotic membranes (experimental) and the other wound is treated with allogenic skin graft (standard of care). All other wound area is treated according to the regular standard of care. During study visit 1 the coverage with human amniotic membranes and human skin allografts will take place in coordination with the eye bank. Following points will be part of the first study visit: photographic documentation, assessment of safety parameters, extraction of laboratory and vital parameters.

### 4.4 Study visit 2: Autologous skin grafting

Visit 2 will take place 3–14 days after visit 1. Autologous split thickness skin grafting will be performed according to preexisting standard operating procedures. The skin grafting will be performed in the operating room. Following will be performed additionally: photographic documentation and the assessment of safety parameters.

### 4.5 Study visit 3: First dressing change after skin grafting

Visit 3 will take place 4–6 days after visit 2 and will mark the first dressing change after skin grafting. The dressing change will be performed by the ICU-staff (intensive care unit)/normal ward staff. Following points will be part of the first study visit: photographic documentation, assessment of primary and secondary outcomes, assessment of safety parameters, extraction of laboratory and vital parameters.

### 4.6 Study visit 4: Dressing change after 12–14 days after skin grafting

Visit 4 will take place 12–14 days after visit 2. The dressing change will be performed by the ICU-staff/normal ward staff. Following points will be part of the first study visit: photographic documentation, assessment of primary and secondary outcomes, assessment of safety parameters, extraction of laboratory and vital parameters.

### 4.7 Visit 5: 90 days postoperatively ( ± 10 days)

Visit 5 will take place approximately 90 days ( ± 10 days) after application of the amniotic membrane and the skin allograft have been applied. This study visit will be part of a routine clinical follow up, but these additional study specific documentations will be performed: photographic documentation, assessment of safety parameters and the clinical assessment of the scar formation using the POSAS-Score.

## 5 Statistical methods

### 5.1 Hypothesis

The null hypothesis of this trial is that amnion and skin allograft lead to the same amount of skin graft percentage take. The investigated alternative hypothesis is that the amnion allograft leads to a greater proportion of skin graft percentage take than the skin allograft.

### 5.2 Determination of sample size

A number of 30 patients was estimated to be required for the trial to have a 90% power to show an absolute difference of 5% points in wound healing at a two-sided alpha level of 0.01, given a paired design with both amnion and skin allograft applied to the same patient, and assuming a wound healing standard deviation of 6.7% points [Muhammadi et al. Burns 2012] [1].

### 5.3 Statistical criteria for termination of trial

The pre-planned study size of 30 patients is final, no interruption of the trial based on statistical criteria is planned.

### 5.4 Planned analysis

Data processing including data entry, cleaning and verification, and preparation of data sets for statistical analysis will be performed locally. In order to ensure uttermost objectivity during the analytical stage, the primary and principal secondary analyses will be conducted with concealed intervention allocation for the statistician, which will only be broken once the analysis is finalized.

Time points chosen for statistical inference of the primary endpoint will be the intervention start point defined as visit 1 and the time points visit 3 at 4–6 days and visit 4 at 12–14days after intervention start. Differences on the primary outcome measure between time points and intervention groups will be tested using linear mixed effects model analysis. As independent variable fixed effects, time point and intervention arm will be entered into the model, respectively. Interaction terms will be tested and included if they add to the models informativity. As random effects, intercepts for subjects as well as per-subject random slopes for the effect of time will be employed, if the model does not suffer overfitting from the addition of the latter. P values will be calculated using Satterthwaite's method of approximation.

Population characteristics will be compared using a paired *t*-test – when continuous – and McNemar's test when categorical. A two-sided p < 0.05 was considered statistically significant. Values will be given as means and 95% confidence intervals or counts and (percentages) as appropriate. Statistical analysis will be performed through a fully scripted data management pathway using the R environment for statistical computing.

### 5.5 Monitoring

Monitoring visits at the investigator's site prior to the start and during the course of the study will help to follow up the progress of the clinical study, to assure utmost accuracy of the data and to detect possible errors at an early time point. All original data including all patient files, progress notes and copies of laboratory and medical test results will be available for monitoring. The monitor will review all or a part of the eCRFs and written informed consents. The accuracy of the data will be verified by reviewing the above referenced documents. The investigator's site will collaborate with the Clinical Trials Center (CTC) of the University Hospital Zurich to ensure monitoring.

## 6 Discussion

The treatment algorithm after burn injuries includes early removal of avital tissue in burn wounds to ensure optimal healing responses [40]. Surgical or enzymatic debridement are feasible and safe approaches to remove the necrotic tissue consequently needing coverage by wound dressings [[40], [41], [42]]. Advanced wound dressings have become a crucial aspect of burn care with a wide range of available dressings with diverse features [43,44]. The ongoing pursuit of the ideal burn wound dressing has led to experiments aimed at optimizing unwanted sequalae, such as infections or delayed re-epithelialization [43,45]. In these experiments the use of hydrogels, honey-based dressing, vacuum therapy, acellular fish skin graft and tissue-engineered constructs, and many more have aimed at challenging split skin grafting as the mainstay of treatment in burn injuries [46].

The use of amniotic membranes in burn treatment dates back as far as 1912 [28]. Since then, numerous studies have been able to explore the beneficial effects of using amniotic membranes as burn wound dressings, leading to an acceleration of the healing process and aiding in the reduction of bacterial colonization of these critical wounds [[29], [30], [31], [32], [33]]. The summative evidence on advantageous effects of treating burns with amniotic membranes have a wide range: lower rates of infection, a significant reduction in pain before and after dressing changes, reduction of hypertrophic scar formation, shorter duration of skin graft take, reduction in burn wound healing time, a reduction of needed dressing changes, a reduction in itching experienced and importantly a low immunogenicity [1,2,4,14,29,31,[47], [48], [49], [50]]. In terms of clinical application, the easy handling, light weight and elasticity give it ideal properties for the use even in complex anatomical areas [4].

Main possible adverse effects are concerning the transmission of infectious diseases such as human immunodeficiency virus, hepatitis C virus, or hepatitis B virus [1,14]. There have been no reports of disease transmissions up until now, given the rigorous screening methods when harvesting the amniotic membranes [30]. Therefore, we expect minimal risks for patients participating in this study since the use of amniotic membrane has proven to be a safe transplant product and we intend to fulfill all necessary safety requirements of Good Manufacturing Practice - as specified in chapters 5, 8 and 18 of the “Guide to the quality and safety of tissues and cells for human application” by the European Directorate for the Quality of Medicines & Healthcare - to avoid any microbiological contamination [51,52].

Reliable additional information on the treatment of burn wounds with amniotic membranes is needed. The knowledge gained in this project may help to routinely include this advantageous modern concept of biological dressings in clinical practice. Moreover, our study will add to the already existing body of knowledge when using amniotic membranes, which have been harvested and processed in an in-hospital supply chain. Current studies have either used commercially available dehydrated amniotic membranes, or as we suggest here, have harvested and processed the membranes in hospital [14,[53], [54], [55]].

The purpose of this study is to use human amniotic membranes, manufactured in our hospital eye bank laboratory, as an allogenic biological dressing after enzymatic debridement in mixed or deep partial thickness burn wounds. Our long-term goal is the implementation of the treatment with amniotic membranes as a standard of care with an in-hospital supply chain. We believe that this will provide a clinically and economically alternative to allogenic skin grafts.

## Funding

This project was funded by the innovation pool from the University Hospital Zurich, Switzerland.

## Data availability

No data was used for the research described in the article.

## Declaration of competing interest

The authors declare that they have no known competing financial interests or personal relationships that could have appeared to influence the work reported in this paper.
